# Hospital Funds and the Working Classes

**Published:** 1887-01-22

**Authors:** Alderman Fred. R. Spark

**Affiliations:** Leeds


### Hospital Funds and the Working Classes.

By Alderman Fred. R. Spark, Leeds.

(*Concluded from page 245.*)

**The Origin of the Leeds Movement.**

In giving a sketch of the movement set on foot in Leeds, to which allusion has already been made, it must not be thought that the working classes of the town have hitherto been more reticent in their gifts to the Local Medical Charities than their fellows in other towns. As a matter of fact, it should be stated that, within the confines of the borough of Leeds, the work-people last year subscribed, in their workshops, the round sum of £2,000 in aid of the three Medical Charities. The population of Leeds may be taken at 334,000, and it is calculated that 80,000 are weekly wage-earners. Sixpence per head per year, or a halfpenny per month, would realise just the amount which last year was collected from the work-people of Leeds for the Medical Institutions. In considering this state of affairs, it occurred to me that this sum was wholly inadequate as representing the ability of the working-classes to contribute towards the maintenance of institutions from which great personal benefit was derived by them. If, by an organisation to reach every workshop in the borough, every weekly wage-earner could be induced to give an average of but a halfpenny per week, then the contributions would reach £8,000 per year, instead of only £2,000. To establish such an organisation as would cover the whole and every minute part of Leeds, so that not a single hand-worker should be free from the opportunity of giving, regularly, a mite for the Medical Charities' Fund, was regarded by some to whom I named my scheme as "a wholly chimerical idea."

Whilst, however, in possession of this idea, the opportunity arose for action. In March last, the annual meeting of the donors and subscribers to the Leeds General Infirmary was held. At the close of the ordinary business I boldly launched my scheme, and asked for the appointment of a sub-committee to consider the subject. This was seconded and adopted, though, it must be confessed, many of those who were present at the meeting looked dubiously at the proposal—especially remembering that other plans having a similar object in view had been tried, and had failed. At the first meeting of this special committee, it was decided to hold a conference with several well-known local leaders of the working-classes. At this conference, the workmen entirely approved of the movement, and gave it as their opinion that the comparatively small amount given by the work-people to the Medical Charities, arose not from a want of will to contribute, but from the want of opportunity. This statement contained the solution of the contemplated plan. It was also made clear that many of the existing modes of gathering in work-people's contributions, both in Leeds and elsewhere, were unreliable. Annual Hospital Saturday collections; donations at any time when fancy or convenience may suggest; collecting-boxes, etc., were discussed; but, as thoroughly effective plans, they were all equally and emphatically condemned. Regular weekly or fortnightly collections of pence in every work-place, large and small, was declared by the work-people's representatives to be the only plan that could succeed, as such a movement ought to succeed.

A Committee Appointed.

The one chief point kept always in view is that of permanency, and the best way to obtain it is by making the contributions as little irksome as possible, and, by regularity of collections, forcing a habit of continuous giving upon the people. Thoroughly to carry this out, it was felt necessary to establish an organisation large and energetic—sufficiently large to cover the whole of Leeds, and sufficiently energetic to carry on the work every week, or, at most, every fortnight. To call a public meeting of work-people for the discussion of the movement, was the next step. This, too, was eminently successful and encouraging. Several of our leading citizens attended, by direct invitation, and delivered addresses. Nor were the workmen one whit behind in their earnest advocacy of the cause. The names of willing workers were solicited, and sixty-three were handed in. This formed the nucleus of the grand committee which is now formed. The scheme developed as it went on, and a great many additional names were at various times sent to the honorary secretaries. It was found possible, after many meetings of the committee, to form a separate committee for each of the sixteen wards into which the borough is divided. Each of these committees, when sufficiently large, met and elected a chairman and secretary; and it was determined that the chief executive should comprise the chairman and secretary of each ward, together with a few other persons representing the various medical institutions. On every hand we have met with willing help. The Mayor has placed his private rooms at our disposal for evening meetings of the executive committee; and the School Board has lent, gratuitously, schools in the various wards for public or other meetings. In nearly every ward public meetings have been held, and at these new names of committee men have been volunteered, until we can now boast of about 400 men who have become workers in the cause.

Further Developments.

Apart from and beyond the financial advantages which will undoubtedly arise from this scheme, there are other advantages, and these have been prominently brought out during the meetings held on the subject. The movement gives an almost unique opportunity for all classes, parties, and sects to meet in furtherance of a common object. Employers and the employed, rich and poor, Churchmen and Dissenters, Tories and Radicals, Home Rulers, Unionists, Separatists, and Dissentionists (or by whatsoever name present-day political parties may be known), all meet together and vie with each other in furtherance of a broad charitable purpose. Working-men at these meetings speak with heartfelt gratitude of the benefits they or their fellows have received from the medical institutions. They laud the skill of the physicians and surgeons, the kind, untiring attention of the nurses, the excellence of the food, and the general management. Gratitude is not an unknown quantity amongst the working-classes. Let me give one instance out of many that have been shown at these meetings. A workman, supporting the resolution in favour of obtaining regular weekly contributions from workshops, told his audience how, for many years, he had been a great sufferer, in consequence of some internal disease. For an average of nearly three months out of every twelve, he was unable to work. He had every year spent a large proportion of his income on medical attendance and medicine. He had become almost weary of his life. At length he was persuaded to go into the Leeds Infirmary, where, after examination and consultation by the medical men, it was determined to operate upon him. This operation was carried out in the most skilful and merciful manner. "And now," the man continued, "for two years I have been in excellent health, and without pain." Probably the recollection of former suffering, and his release therefrom, affected the poor fellow so much that he was quite overcome, and, weeping, he resumed his seat. The cheers for him that went up from the working-men present were eloquent in their sympathy, and showed how "one touch of nature makes the whole world kin."

Other Advantages.

These meetings, too, have had another good effect. They have aroused among the well-to-do classes a greater interest in the medical charities. Instances have become known where subscribers have increased their subscriptions, and new subscribers have been found, as direct consequences of the publicity given to the movement. A manufacturer at one of the meetings volunteered the statement that up to the present time he had not been an annual subscriber to the medical charities. "But how," said he, "can I ask the working-men to give regularly, if I don't set the example? So I have sent in my name as an annual subscriber."

The Well-to-do Classes.

The real work of the organisation—that of collection

**The Crucial Test.** —was begun on January 1st; and, of course, it remains yet to be seen how far the plan will succeed. The following hand-bill has been circulated by thousands, and its contents will convey information which may be useful to any town desirous of emulating what has been done in Leeds :—

### LEEDS MEDICAL CHARITIES.

### WORK-PEOPLE'S COMMITTEE.

*Chairman* . . . THE MAYOR OF LEEDS.

The object of this movement is to extend Collections among the Work-people of the Borough of Leeds in aid of the Three Medical Charities, viz. : The Leeds General Infirmary, The Leeds Dispensary, The Leeds Hospital for Women and Children.

It is calculated that three-fourths of the Leeds Work-people have not hitherto subscribed to the Medical Charities. To induce these to give their mite, a Committee has been formed in each of the Sixteen Wards of the Borough, which Committee will assist Workshops, Factories, Mills, and other places to obtain Small Weekly or Fortnightly Sums in aid of the Charities.

Numerous meetings of Work-people have recently been held in Leeds, and every step has been taken to ascertain their views as to the mode of collection, and the distribution of the funds. The following Modes of Collection have been proposed :

1. A Collector to be appointed by the Work-people who shall, Weekly or Fortnightly, gather from everyone employed in the Works a Halfpenny or a Penny, according to the position of the Worker.
2. Collectors in the various Departments to pursue the same plan.
3. The giving of work as overtime—the value to be paid to the Medical Charities' Fund.
4. An arrangement with the Employer by which the Cashier, or Wage-payer, may deduct from the wages of each person a Halfpenny or a Penny every week.

It is intended to establish one General Fund, and to divide this Fund between the three Medical Institutions in the same proportion as the sums given last year by the Work-people of Leeds. This division has received the assent of the three Charities.

In addition to Collections from Work-places, it is proposed to gather regular subscriptions from Shopkeepers and Home-workers.

Also, it has been arranged to hold an Annual Leeds Hospital Gala in Roundhay Park—the first to take place on *Saturday, July 2nd*, 1887.

It is urged that all monies collected, whether in Work-places or by the Ward Committees, be handed over to the Honorary Treasurer at the Infirmary every month, or certainly not at a longer period than quarterly.

For Collections in Shops and from Home-workers, an official receipt-book will be provided, and it is expressly arranged that for all amounts so collected, a printed receipt shall be given.

R. BENSON JOWITT, *Hon. Treas.*

FRED. R. SPARK, } *Hon. Secs.*

T. BALLAN STEAD, }

LEEDS, *January 1st*, 1887.

Should we succeed in doubling the ordinary contributions of the work-people in this town, and thus add £2,000 a year more to the funds of the medical charities, an organisation will have been established which may lead to benefits incalculable.

### EPITAPH ON A DOCTOR.

"*His end was patients.*"

